# Impact of culture dimensionality and matrix composition on morphology, phenotype and drug response in pancreatic cancer models

**DOI:** 10.1038/s41598-026-47856-1

**Published:** 2026-04-14

**Authors:** Florian Doelvers, Katharina Wansch, Anna Kuehn, Mihnea P. Dragomir, Tobias Janik, Maria Joosten, Georg Hilfenhaus, Loredana Vecchione, Matthäus Felsenstein, Dou Ma, Markus Lerchbaumer, Christian Jürgensen, Marcus Bahra, Gregor Duwe, Sebastian Stintzing, Ulrich Keilholz, Uwe Pelzer, Christopher C. M. Neumann

**Affiliations:** 1 Department of Hematology, Oncology and Tumor Immunology, Charité-Universitätsmedizin Berlin, Freie Universität Berlin, Humboldt-Universität zu Berlin, Berlin Institute of Health, Berlin, Germany; 2 Department of Pathology, Charité-Universitätsmedizin Berlin, Freie Universität Berlin, Humboldt-Universität zu Berlin and Berlin Institute of Health, Berlin, Germany; 3 German Cancer Consortium (DKTK), Partner Site Berlin and German Cancer Research Center (DKFZ), Heidelberg, Germany; 4 Berlin Institute of Health (BIH), Berlin, Germany; 5 Department of Surgery, Charité-Universitätsmedizin Berlin, Freie Universität Berlin, Humboldt-Universität zu Berlin and Berlin Institute of Health, Berlin, Germany; 6 Department of Radiology, Charité-Universitätsmedizin Berlin, Freie Universität Berlin, Humboldt-Universität zu Berlin and Berlin Institute of Health, Berlin, Germany; 7 Department of Hepatology and Gastroenterology, Charité-Universitätsmedizin Berlin, Freie Universität Berlin, Humboldt-Universität zu Berlin and Berlin Institute of Health, Berlin, Germany; 8 Department of Surgical Oncology and Robotics, Krankenhaus Waldfriede, Lehrkrankenhaus der Charité, Berlin, Germany; 9 Department of Urology and Pediatric Urology, University Medical Center Johannes Gutenberg University, Mainz, Germany; 10 Charité Comprehensive Cancer Center, Charité – Universitätsmedizin Berlin, Berlin, Germany

**Keywords:** Pancreatic Ductal Adenocarcinoma, Patient-Derived Models, 2D-3D Cell Culture, Basement Membrane Extract, Chemoresistance, Drug Sensitivity Testing, Cancer, Oncology

## Abstract

Patient-derived in vitro systems are powerful preclinical models that replicate key features of human tumors and enable investigation of cancer biology and drug response. They are particularly promising for pancreatic cancer research. We examined whether culture dimensionality and basement membrane extract (BME) composition affect establishment rates, morphology, proliferation, marker expression, and drug response in patient-derived models of pancreatic ductal adenocarcinoma (PDAC). From 12 PDAC samples, matched two-dimensional primary cell lines (PDCLs) and three-dimensional organoids were established in Cultrex (PDOCs) and Matrigel (PDOMs). PDCLs formed monolayers, while PDOCs and PDOMs developed cystic or dense organoids, independent of BME type. Immunohistochemistry showed no differences in key diagnostic markers between culture systems, and Ki-67 levels were consistently higher in vitro compared to original tumors. Pharmacological testing with five standard chemotherapeutics revealed no significant differences in drug response between dimensionalities or BMEs, although 3D models were detected to be slightly more chemoresistant. In two patients treated with gemcitabine monotherapy, in vitro therapy response correlated with clinical relapse. Our findings indicate that while drug responses are largely patient-specific and independent of dimensionality and matrix composition, 3D models more realistically recapitulate tumor architecture and phenotypes, supporting their value for translational PDAC research.

**Trial registration:** The study was conducted according to the guidelines of the Declaration of Helsinki. Ethical approval was requested and granted by the ethics committee of the Charité Universitätsmedizin Berlin (EA1/157/21) on 26th May 2021.

**Supplementary Information:**

The online version contains supplementary material available at 10.1038/s41598-026-47856-1.

## Introduction

Pancreatic ductal adenocarcinoma (PDAC) is among the most lethal malignancies, with a five-year survival rate of only 10%^1^. The high mortality can be explained by unspecific symptoms, rapid metastatic spread, aggressive tumor biology and intrinsic resistance to standard therapies such as mFOLFIRINOX (5-fluorouracil, calcium folinate, oxaliplatin, irinotecan), gemcitabine plus nab-paclitaxel and gemcitabine monotherapy^2–4^. Preclinical in vitro models are essential to improve our understanding on the PDAC biology and can help to guide translational research. Since the establishment of the first PDAC cell line (PANC-1) in 1975^5^, numerous two-dimensional (2D) cell lines have been developed for mechanistic studies, drug screening and molecular profiling. These models are cost-effective, reproducible and compatible with standard culture media such as RPMI^6–8^. Establishment rates for primary PDAC cell lines in 2D vary, ranging from 7% to 79%^9–11^. In 2D, cancer cells adhere to flat plastic surfaces, leading to unnatural morphology, anchorage independence, loss of polarity and altered receptor distribution, all of which can affect drug response^8^. Furthermore, the absence of extracellular matrix components impairs physiological cell-cell and cell-matrix interactions that are critical for regulating survival and drug resistance pathways. This is particularly relevant for PDAC, as the tumor microenvironment and stroma can account for up to 90% of the tumor volume^12^. In 2D cultures, cells are uniformly exposed to nutrients and compounds due to their monolayer structure, which can further increase drug efficacy^8,13–16^.

To address these limitations, three-dimensional (3D) systems such as tumor spheroids and patient-derived organoids (PDOs) have been introduced^17^. PDOs are typically embedded in basement membrane extracts (BMEs) and cultivated with growth factors like Wnt, nicotinamide and human noggin to achieve high establishment rates of up to 83%^17–19^. BMEs such as Matrigel and Cultrex, both derived from Engelbreth-Holm-Swarm (EHS) sarcomas, support 3D growth which better replicates in vivo tumor biology^8,17,20^. Previous studies suggested that pharmacotyping and transcriptomic profiles of PDOs are largely consistent across different matrices^8,20^. Due to their ability to better mimic in vivo tumor cell physiology, organoid-based models have gained increasing regulatory attention and are now considered by the U.S. Food and Drug Administration (FDA) as potential alternatives to animal testing for evaluating drug toxicity^21^.

In this study, we systematically investigated how culture dimensionality and matrix composition influence establishment rates, morphology, proliferation, immunohistochemical marker expression and chemotherapeutic response in patient‑derived PDAC models. From 12 resected PDACs, we attempted to isolate matched 2D patient-derived cell lines (PDCL), PDOs in Cultrex (PDOC) and PDOs in Matrigel (PDOM). A complete series was achieved in three cases as well as a single case of matched PDOC and PDOM models. We assessed morphology, proliferation dynamics, immunophenotype and in vitro therapy response to 5-fluorouracil, oxaliplatin, SN-38, gemcitabine and paclitaxel. We also correlated gemcitabine response with relapse in two patients.

## Results

### Choice of 3D matrix

To ensure methodological relevance, we performed a systematic literature search focusing on published human PDAC patient-derived organoid (PDO) models from the past ten years. Among 65 eligible studies describing patient-derived establishment of epithelial PDAC organoids, Matrigel was used in 90.8% of studies, while Cultrex was used in 9.2% (including exclusive and combined usage with Matrigel). Alternative matrices, such as collagen-based systems, have only been reported sporadically. In the present study, we deliberately refrained from using collagen-based matrices, as our focus was specifically on the epithelial compartment of PDAC. These findings demonstrate that Matrigel and Cultrex represent the two most commonly used extracellular matrices in PDAC PDO research.

### Characterization of patient cohort

For this study, a total of 16 patients with suspected PDAC in 2023 were included. Histopathologically PDAC was confirmed in 12 cases. The remaining 4 samples were histologically confirmed neuroendocrine carcinoma (NEC, *n* = 1), medullary-type PDAC (*n* = 1), papillary adenoma (*n* = 1) and a benign lesion (*n* = 1). The median age of the patient cohort was 66.9 years and the male to female ratio was 3:9 in the PDAC patient cohort. According to UICC staging, 58.4% of tumors were classified as stage II and 33.4% as stage III. One patient was pre-treated, 11 patients were therapy-naive (no pre-treatment). All tissue samples were derived from surgical resections. A completed series with all three model types (PDCL, PDOC, PDOM) was achieved in 3 out of 12 cases (25%). In one case PDOC and PDOM model types were simulatenously achieved. Initial, but non-sustained growth in all model types was observed in 5 cases (41,7%) and no growth occurred in 4 cases (33,34%) (Table 1).

**Table 1:** Clinical and experimental characteristics of the study cohort (*n* = 12). Data are presented as absolute numbers and percentages. Median age is given with range.

Characteristics	Total (*n*, %)
Number of patients Median age on the day of surgery	12
	66,9 (52–77) years
Sex	
Male	3 (25)
Female	9 (75)
Pretreatment	
mFOLFIRINOX	1 (8,4)
No treatment	11 (91,6)
UICC	
I	1 (8,4)
II	7 (58,4)
III	4 (33,4)
IV	0
Sample type	
Surgical sample	12 (100)
In vitro growth	
No growth	3 (25)
Initial growth	5 (41,7)
Established PDCL	3 (25)
Established PDO Cultrex	4 (33,4)
Established PDO Matrigel	4 (33,4)
Established in all conditions	3 (25)

DNA sequencing was performed on PDOCs generated from each patient. In all three PDOC models, activating mutations in *KRAS* were detected (PDO Patient 1: *KRAS* p.G12V (AF 99%); PDO Patient 2: *KRAS* p.G12V (AF 68%); PDO Patient 3: *KRAS* p.G12C (AF 100%)). In addition, alterations in *TP53* were identified (PDO Patient 1: *TP53* p.S96fs (AF 98%); PDO Patient 2: *TP53* p.E298* (AF 98%); PDO Patient 3: *TP53* p.P151S (AF 99%)). In PDO Patient 4, no mutations in KRAS or TP53 were detected. Instead, variants were identified in SOX9 (p.M218T, AF 54.5%), KMT2C (p.H2489P, AF 47.4%), CD274 (p.H233L, AF 46.3%), and GLI1 (p.P9Q, AF 4.4%). These findings verify the malignant origin of the in vitro models and confirm the presence of PDAC driver mutations.

### Characterization of growth kinetics, immunohistochemical marker expression and morphological behavior

**Fig. 1:**
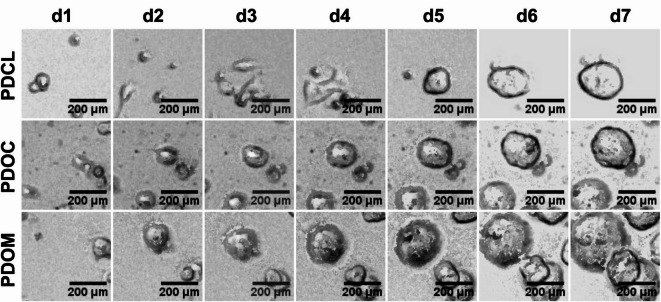
Brightfield microscopy of patient-derived PDAC cultures over 7 days. Representative brightfield images of PDCL, PDOC and PDOM models derived from patient 3. Cultures were imaged at days 1, 2, 3, 4, 5, 6 and 7 (columns) using confocal microscopy in brightfield mode. PDCLs exhibited dispersed, irregular cell growth with limited clustering. In contrast, PDOC and PDOM cultures developed progressively larger and more defined organoids over time, with similar morphology and structural density in both matrix conditions. Scale bars = 200 μm.

Morphological profiling revealed distinct growth patterns between 2D and 3D culture systems. PDCL cultures exhibited two-dimensional monolayer growth, typically forming small, loosely associated clusters of cells adherent to the flask surface. The majority of cells remained attached and floating cells were rarely observed. Cluster building of cells was minimal. In contrast, both PDOC and PDOM cultures developed progressively larger and more compact organoids from days 1 to 7, displaying continuous growth, increasing organoid size and density. Organoids formed in both Cultrex and Matrigel matrices exhibited either cystic or dense organoid morphology. The type of organoids formed was patient-specific: two cultures developed exclusively cystic structures, one developed dense organoids and one exhibited both morphologies within the same culture conditions. No major morphological differences were observed between PDOC and PDOM (Fig. 1). To further assess potential differences in PDO size between matrix conditions, diameters of all visible organoids within standardized brightfield confocal fields of view were quantified at day 3 and day 7. Mean PDO diameters were comparable between PDOC and PDOM cultures at both time points, with mean diameters of 108 ± 66 μm (PDOC) and 112.5 ± 83 μm (PDOM) at day 3 and 166.5 ± 116 μm (PDOC) and 175.5 ± 134 μm (PDOM) at day 7. To further assess whether organoid morphology reflects the architecture of the corresponding primary tumors, a direct histomorphological comparison was performed using H&E-stained sections of FFPE primary tumor tissue and matched organoids (PDOC/PDOM). All gland-forming G2 primary tumors (Patients 1, 2, and 4) exhibited well-defined epithelial glandular structures in the FFPE as well as strong stromal compartments^22^. The corresponding organoids similarly displayed glandular architectures, including mono- and multilayered epithelial gland formation, independent of the extracellular matrix used (Cultrex or Matrigel). In contrast, the poorly differentiated G3 tumor (Patient 3), characterized by solid growth and tumor nests in the FFPE specimen, generated organoids with predominantly compact, nest-like and cribriform architectures rather than cystic glandular structures. The detailed findings are provided in Supplementary Table S1.

**Fig. 2:**
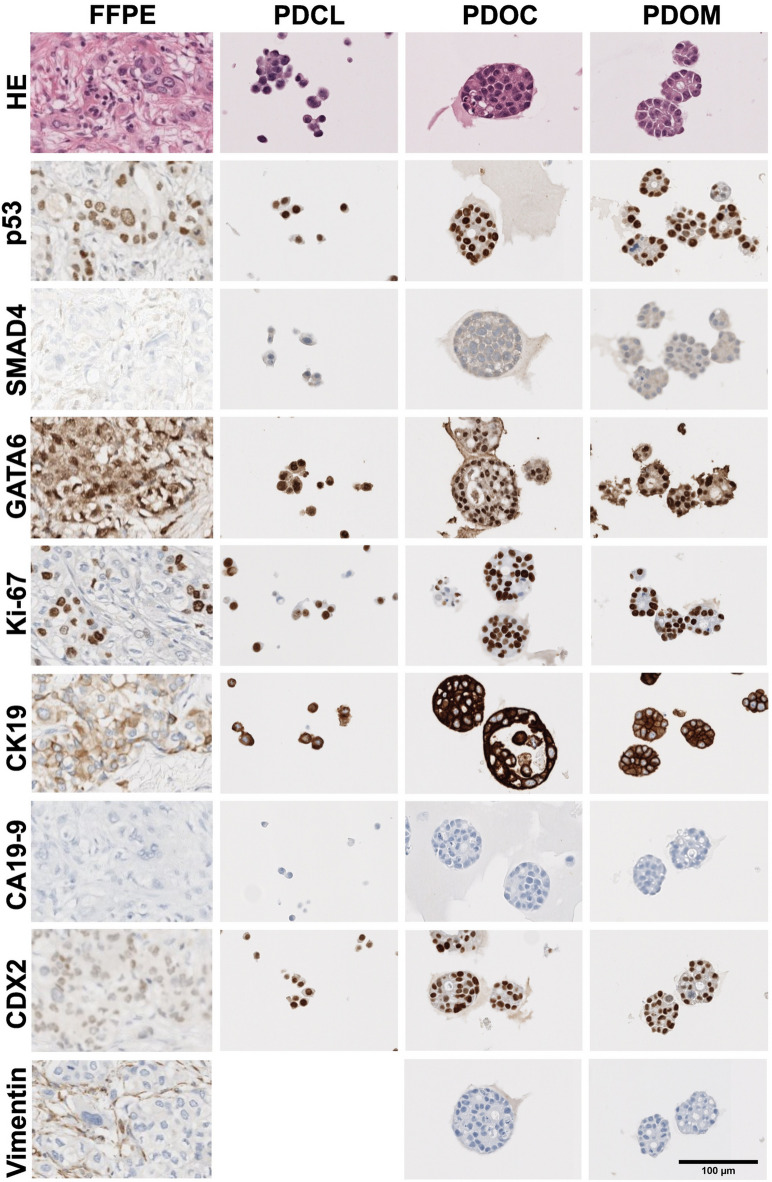
Histological and immunohistochemical staining of matched FFPE tissue, PDCL, PDOC and PDOM from a representative PDAC patient. Hematoxylin and eosin (HE) staining and immunohistochemistry (IHC) were performed on matched FFPE tissue, PDCL, PDOC and PDOM. Nuclear markers (p53, Ki-67, GATA6, SMAD4, CDX2) and cytoplasmic or membranous markers (CK19, CA19-9, Vimentin) were assessed to compare marker expression profiles across culture models. Scale bar = 100 μm.

**Fig. 3:**
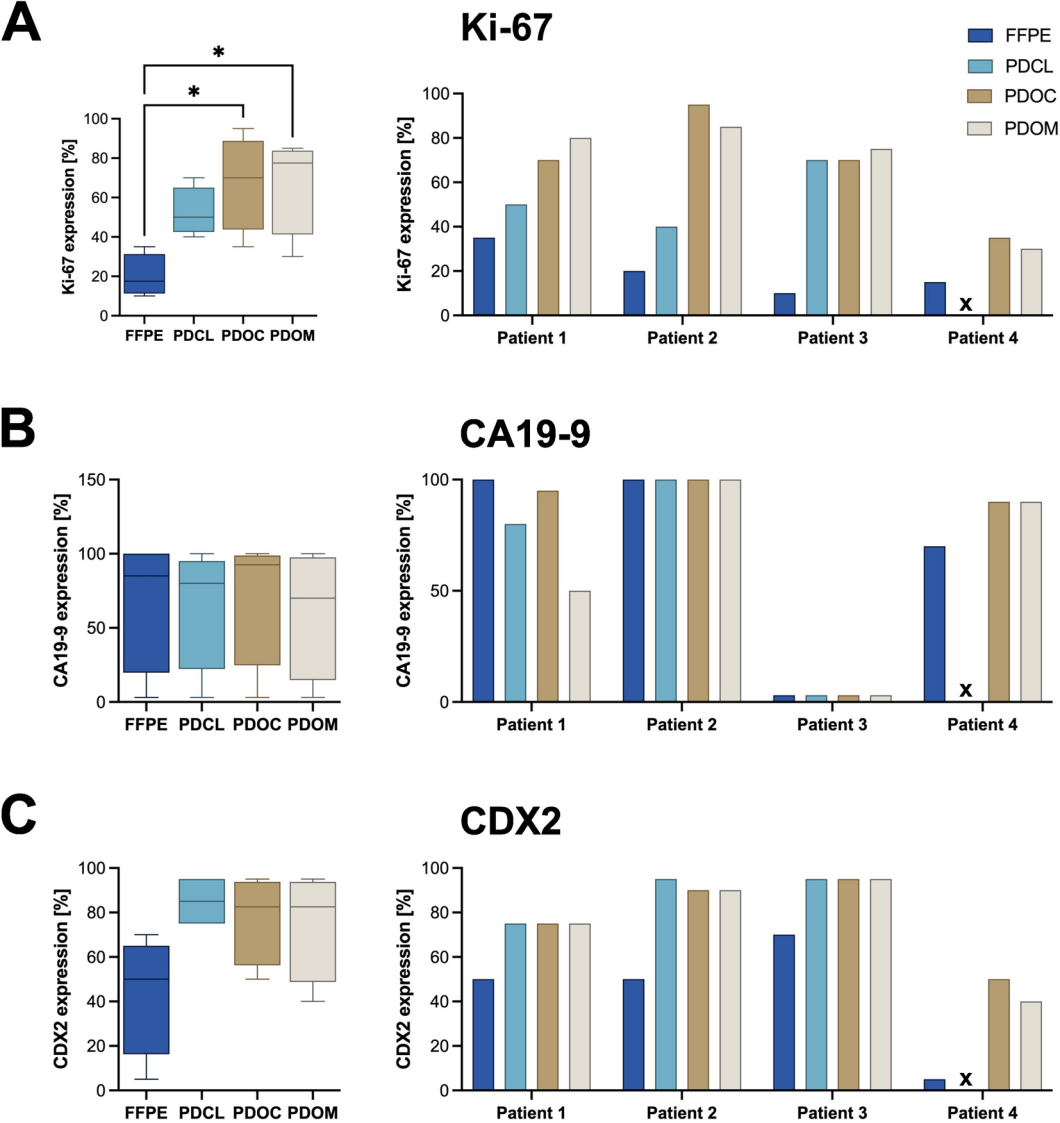
Quantitative comparison of Ki-67, CA19-9 and CDX2 expression between FFPE tumor tissue and matched in vitro models. (**A**) Left: Box plot showing Ki-67 expression (%) across all samples (*n* = 4) in FFPE tissue, PDCL, PDOC and PDOM cultures. Ki-67 expression was significantly higher in 3D cultures compared to FFPE (*p* < 0.05). Right: Bar plots display individual patient values. (**B**) Left: Box plot of CA19-9 expression showing no significant differences between FFPE and in vitro models. Right: CA19-9 expression per patient. (**C**) Left: CDX2 expression (%) non significant higher in in vitro models compared to FFPE, particularly in PDCL cultures. Right: CDX2 expression per patient.

Apart from Ki-67 and CDX2, immunohistochemical analyses revealed largely preserved markers when comparing the FFPE tissue and the different in vitro culture models (Fig. 2). On average, Ki-67 expression was elevated in in vitro cultures compared to the FFPE tissue (52,5% PDCL, 67,5% PDOC and PDOM, 20% FFPE, Figs. 2 and 3A). CA19-9 expression levels were comparable across conditions, with high variability between patients - particularly low expression in patient 3 and negative stainings in the derived in vitro cultures (Fig. 3B). CDX2 expression was elevated in all culture conditions compared to FFPE, especially in PDCLs (Fig. 3C). p53 and SMAD4 staining patterns in vitro generally mirrored the tumor-specific mutational status. GATA6 and CK19 were robustly expressed in all conditions, confirming preservation of ductal epithelial differentiation. Vimentin staining was predominantly negative or focal positive in vitro (see also Supplementary Table S2), suggesting limited mesenchymal transition across models (Fig. 2).

To assess whether proliferative activity within organoids was associated with organoid size, Ki-67 expression was correlated with organoid diameter measured in Ki-67-stained PDOC and PDOM cultures from all four patients. Spearman correlation analysis revealed no significant association between organoid diameter and Ki-67 expression (Spearman *r* = − 0.19, *p* = 0.0866; *n* = 80). However, the negative correlation coefficient together with a p-value approaching statistical significance indicates a trend toward decreasing Ki-67 expression with increasing organoid diameter (Supplementary Figure S1).

**Fig. 4:**
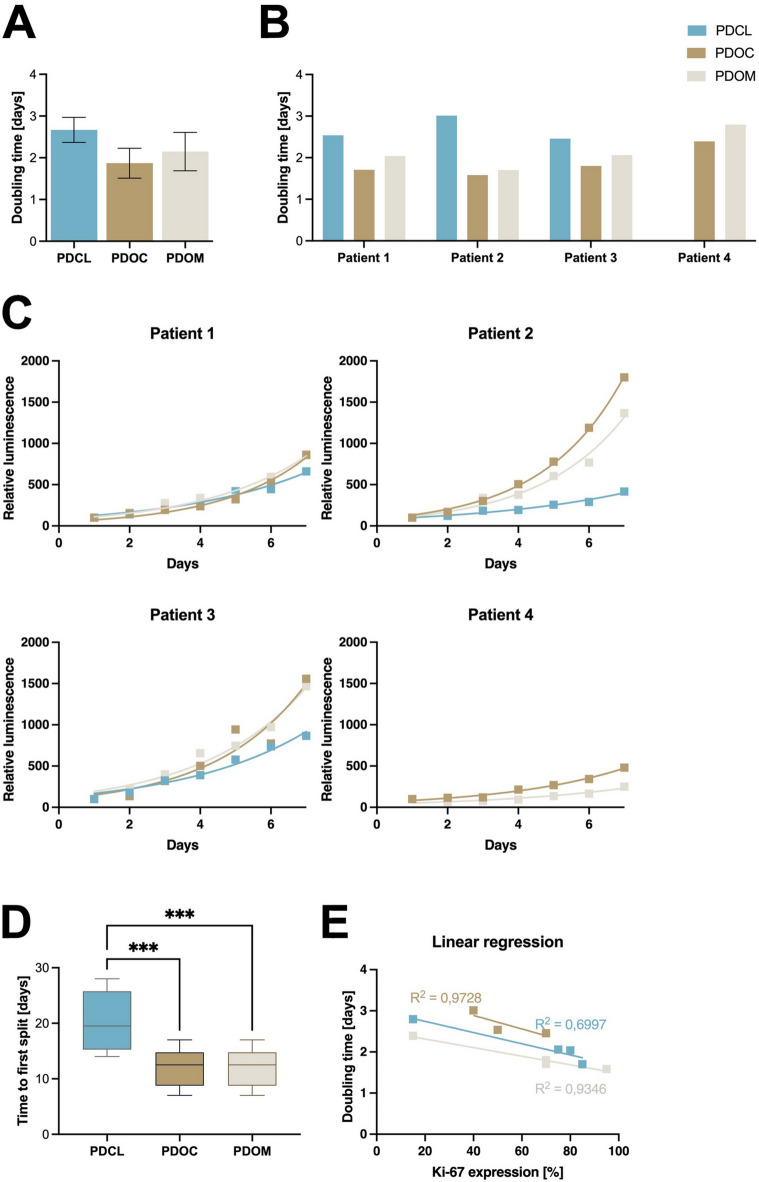
Growth kinetics and proliferation in patient-derived PDAC culture models. (**A**) Comparison of mean doubling times (DT) across culture models (PDCL, PDOC, PDOM). Asterisks indicate statistically significant differences (***p < 0.001). (**B**) Doubling times per patient in each model. (**C**) Growth curves showing relative luminescence over 7 days for PDCL (blue), PDOC (brown) and PDOM (beige) in four patients. (**D**) Time to first split (TTFS) across culture models; ***p < 0.001. (**E**) Linear regression of Ki-67 expression (%) versus DT for each model. Each point represents one patient-derived culture model.

Time to first split (TTFS) was significantly longer in PDCL cultures compared to both PDOC and PDOM (*p* < 0.001; Fig. 1D). On average, TTFS was 8 days longer in PDCL cultures compared to both PDOC and PDOM (mean difference: 8.04 days, 95% CI: 12.42 to 3.67). No significant difference was observed between PDOC and PDOM (*p* > 0.9999). Longitudinal growth curves based on relative luminescence demonstrated exponential proliferation in all models over a seven‑day period (Fig. 4C). Pooled doubling time (DT) analysis showed longer proliferation times in PDCLs compared to PDOC and PDOM (*p* = 0.0636 and *p* = 0.2440 respectively; Fig. 4). No significant difference in DT was detected between PDOC and PDOM (*p* = 0.5898). Moreover, the quantification of proliferation (DT) was linked to the Ki-67 expression observed above. A low Ki-67 in in vitro models was linked to longer DT (PDOC, R² = 0.97, *p* = 0.0137; PDOM R² = 0.93, *p* = 0.0333; PDCL R² = 0.70, *p* = 0.37; Fig. 4E).

### Inter-patient, inter-model comparison of resistance to commonly used cytostatics in PDAC and clinical correlation

**Fig. 5:**
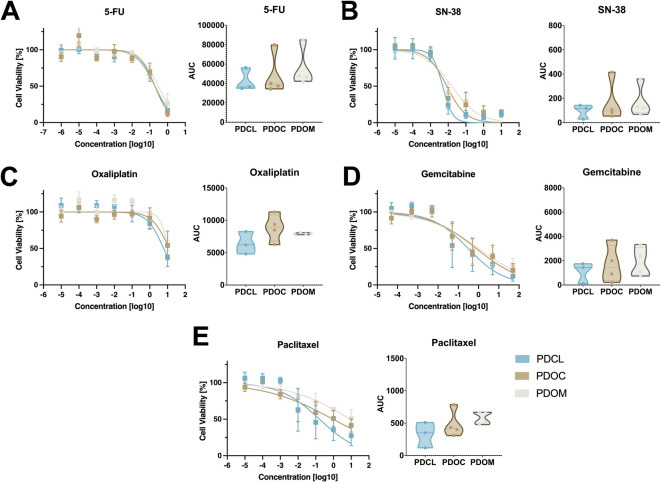
Pharmacological profiling of patient-derived PDAC culture models. (**A–E**) Dose-response curves and area under the curve (AUC) analyses for 5-FU (**A**), SN-38 (**B**), Oxaliplatin (**C**), Gemcitabine (**D**) and Paclitaxel (E) across PDCL (blue), PDOC (brown) and PDOM (beige) models. Left panels: Curves represent the mean of individual patient-derived cultures; error bars indicate standard deviation reflecting inter-patient variability. Right panels: Comparison of AUC values for each culture model. Each point represents an individual patient-derived culture; violin plots illustrate distribution and variability across models.

Pharmacotyping of single agents of the mFOLFIRINOX and gemcitabine/nab-paclitaxel regimens showed consistent cytotoxic profiles across all three culture conditions in inter-model comparisons. These included 5-fluorouracil (5-FU), SN-38, oxaliplatin, gemcitabine and paclitaxel (Fig. 5A–E, left panels). Generally, 3D cultures tended to be slightly more chemoresistant to all agents than their 2D counterparts. Due to the low number of cases, however, this trend did not reach statistical significance. Further, no significant differences in log_10_GR50 values, adjusted for individual DT, were observed between the culture models for any agent (ANOVA all *p* > 0.22). For detailed dose response curves of each in vitro model, see Supplementary Figure S2.

**Fig. 6:**
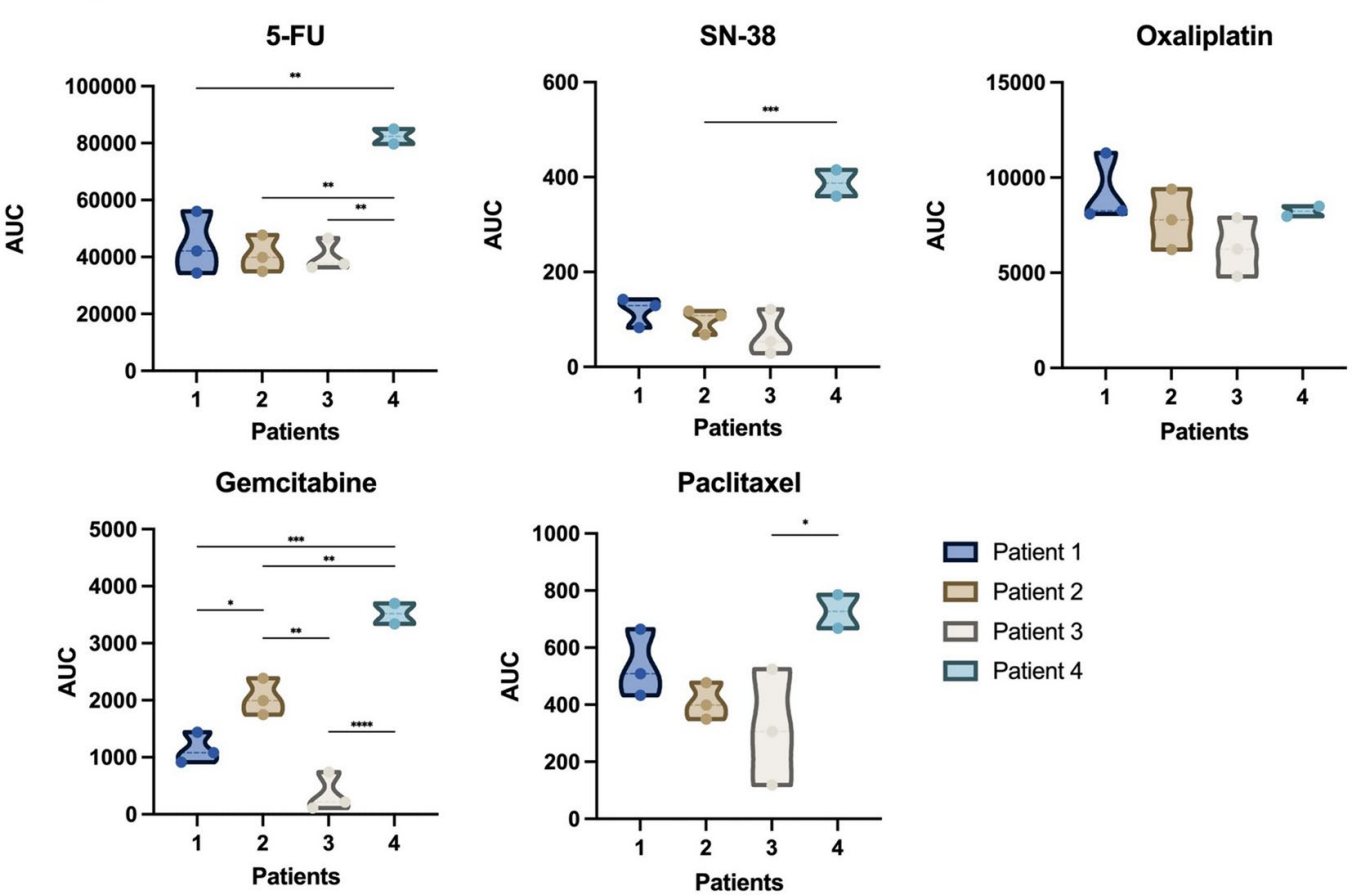
Inter-patient heterogeneity in chemotherapeutic response visualized by AUC distributions. Violin plots depicting the distribution of AUC values derived from dose-response curves for 5-fluorouracil, SN-38, oxaliplatin, gemcitabine and paclitaxel across four PDAC patients. Each violin includes all available in vitro culture models per patient (PDCL, PDOC, PDOM), with individual data points.Statistical significance between patients was assessed by one-way ANOVA followed by post hoc multiple comparisons. Asterisks indicate significance levels: *p < 0.05, **p < 0.01, ***p < 0.001, ****p < 0.0001.

Differing chemotherapeutic response was observed between different patients. AUC values for 5-fluorouracil (5-FU), SN-38, oxaliplatin, gemcitabine and paclitaxel were compared across the four PDAC patients, pooling results from patients for each culture system (PDCL, PDOC, PDOM, Fig. 6). Gemcitabine exhibited the most pronounced variability between patients (*p* < 0.0001). For both 5‑FU and SN‑38, AUC values varied significantly between patients (*p* < 0.05), with multiple pairwise differences observed. No significant inter-patient variability was observed for oxaliplatin (*p* = 0.23).

**Fig. 7:**
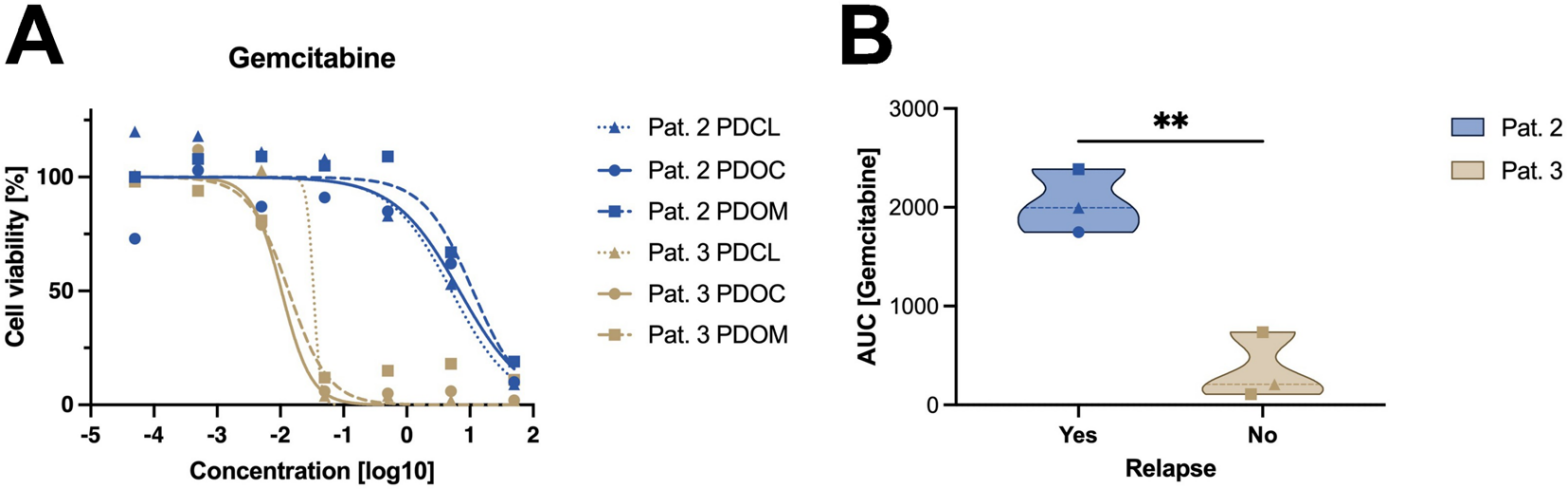
Correlation of in vitro gemcitabine response with clinical relapse. (**A**) Dose–response curves for gemcitabine in matched PDCL, PDOC and PDOM models derived from Patient 2 (blue) and Patient 3 (brown). Cell viability [%] was measured 72 h post-treatment and data are presented as mean of technical replicates. (**B**) Comparison of area under the curve (AUC) values for gemcitabine response in relation to clinical relapse status. Each data point represents the AUC of one culture model. Patient with relapse (Patient 2) exhibited significantly higher AUC values compared to the non-relapsing patient (Patient 3) (*p* < 0.01).

In a final step, the in vitro response was correlated with the clinical course of the respective patients. Patients 2 and 3 underwent R0 resection and received adjuvant monotherapy with gemcitabine. While patient 2 relapsed after 168 days. Patient 3 remained disease-free. Consistently, the AUC of patient 2 was markedly higher than that of patient 3 (Fig. 7), indicating a higher chemoresistance in the cultures derived from patient 2. No significant differences in drug response were observed between the different cell culture systems. To further characterize potential resistance-associated markers, MDR1 expression was assessed by immunohistochemistry using H-scores. The gemcitabine-resistant PDOC model derived from Patient 2 exhibited markedly elevated MDR1 expression with an H-score of 270. In contrast, Patient 3, who showed sustained clinical remission and increased in vitro gemcitabine sensitivity, demonstrated substantially lower MDR1 expression (H-score 100). Patients 1 and 4 displayed intermediate MDR1 expression levels, with H-scores of 115 and 120, respectively.

Furthermore, patients 1 and 4 received adjuvant mFOLFIRINOX. Both patients died within the first 6 months after resection, with the cause of death of one patient remaining unknown. Thus, a relative clinical comparison for mFOLFIRINOX was not feasible for these patients.

## Discussion

In this study, we systematically investigated how cell culture dimensionality (2D vs. 3D) and extracellular matrix composition (Cultrex vs. Matrigel) influence establishment rates, morphology, proliferation, immunohistochemical marker expression and chemotherapeutic response in patient‑derived PDAC models. Another objective was to evaluate the extent to which different culture systems affect the preclinical drug testing in PDAC. Since Cultrex and Matrigel were known to be the most commonly used matrices as presented in our extensive literature research, the experiments focused on the matrices most relevant to the current practice in the field.

Previously reported establishment rates for PDOs range between 63% and 83%^17–19^.Based on an assumed success rate of 65% per system, the calculated combined probability across three parallel systems (0.65³ ≈ 27.5%) is in close agreement with the overall establishment rate observed in our cohort (25%). However, this calculation assumes the probability of establishment to be statistically independent across cultivation systems and largely determined by model-specific factors rather than patient-specific characteristics. In our cohort, this assumption did not hold. Tumor cells typically either grew in all systems or failed to be established in any of them, with only a single exception where growth was not observed in the 2D system. This pattern suggests that patient- or tumor-intrinsic factors, rather than culture conditions, are the dominant determinants of successful culture establishment. In a recent systematic analysis conducted by our group, clinical and immunohistochemical determinants of successful in vitro model establishment from PDAC tissue were investigated. In this study, higher UICC stage, particularly metastatic disease (UICC IV), as well as elevated serum CA19-9 levels and increased GATA6 expression in the source tumor tissue were associated with improved establishment rates of patient-derived in vitro models^23^. Since the cohort of 12 patients included in our current study included resectable, non-metastatic PDAC patients, the establishing rate might have been expected to be lower than previously reported rates. Furthermore, morphological analyses revealed marked distinctions between patient‑derived 2D (PDCLs) and 3D (PDOCs and PDOMs) culture systems. While PDCLs exhibited typical monolayer growth with limited clustering, both PDOCs and PDOMs developed compact and structured organoids, consistent with previously described two- and three‑dimensional tumor models^8,24^. Interestingly, organoid morphology appeared to be patient‑specific, with cystic, dense or mixed morphological organoid types observed independently of the matrix (Cultrex vs. Matrigel), suggesting that intrinsic tumor properties outweigh matrix‑derived effects^20^. This observation was further supported by direct histomorphological comparison with the corresponding FFPE primary tumors. All gland-forming G2 carcinomas gave rise to organoids recapitulating epithelial glandular structures with mono- and multilayered organization, irrespective of the extracellular matrix used. In contrast, the only poorly differentiated G3 tumor, characterized by solid growth and tumor nests in the primary specimen, generated predominantly nest-like and cribriform organoid architectures rather than cystic glandular structures. Together, these findings indicate that patient-specific epithelial architectural traits are preserved in the organoid system and largely reflect the differentiation status of the original tumor. This observation aligns with findings from a previous study which reported organoid morphology to be highly patient-specific^25^. Beyond intrinsic architectural features, organoid size itself may represent an additional factor influencing characteristics within three-dimensional structures. Previous studies have suggested that organoids are subject to oxygen diffusion limits and may develop necrotic cores beyond a critical size threshold, resulting in reduced proliferation in central regions^26^. Although no statistically significant correlation between organoid diameter and Ki-67 expression was observed in the current study, the negative correlation trend in our cohort indicates that larger organoids tend to show lower Ki-67 expression as marker for proliferative activity. This observation may relate to increasing hypoxia and the onset of central necrosis at greater diameters, as described for larger organoid structures^26^.

Proliferation analyses demonstrated that 3D models consistently showed faster growth compared to PDCLs, as indicated by shorter DT and reduced TTFS. This finding is in contrast to previous studies using long‑established 2D cell lines, which are typically immortalized and often exhibit accelerated proliferation compared to PDOs^8^. Clonal selection and genomic divergence during serial passaging have been demonstrated in established two-dimensional PDAC cell lines^27^. In the current study, our aim was to assess differences between patient-derived 2D cultures and PDOs under the same conditions. To this end, PDCLs and PDOs were isolated from the same tumor tissue, cultured in identical media, and were all analyzed within passages 3 to 15. We acknowledge that these conditions may not represent optimized 2D culture systems and that coating plates with laminin or collagen could eventually facilitate adaptation^28^. In our case, the use of early‑passage, patient‑derived cell lines may preserve proliferative restraint characteristic of primary tumor cells. As a known immunohistochemical marker for proliferation, high Ki‑67 expression of PDOs also correlated with shorter DT and reduced TTFS^9^. Moreover, Ki‑67 levels were significantly higher in 3D models compared to matched FFPE tissue, which may reflect further clonal selection of more proliferative tumor cells by growth‑supporting culture conditions^17^.

Furthermore, phenotypic expression levels of p53, SMAD4, CA19‑9, CK19, GATA6 and Vimentin were preserved across all culture models. The strong expression of GATA6 and CK19 as well as the low Vimentin expression confirmed the ductal epithelial characteristics and are characteristic features of the classical PDAC subtype^29–31^. CDX2 is a well-established marker of intestinal differentiation^32^. Interestingly, its expression was elevated in vitro compared to matched FFPE tissue samples. This upregulation may indicate culture-induced shifts in differentiation status or enhanced cellular plasticity.

Pharmacological profiling across standard chemotherapeutics (5‑FU, SN‑38, oxaliplatin, gemcitabine and paclitaxel) revealed no significant differences in drug response between 2D and 3D culture systems in our study. This observation aligns with previous reports showing similar chemoresistance across culture dimensionalities^20,24^. The lack of differences observed for five commonly used chemotherapeutic agents likely reflects their broad, non‑specific mechanisms of action. For example, oxaliplatin exerts its cytotoxic effect by forming DNA crosslinks and thereby inducing apoptosis irrespective of specific signaling pathways^33^. In contrast, Peschke et al. reported differential responses between matched 2D and 3D cultures derived from the same tumor to the MEK inhibitors benimetinib and cobimetinib, which target the MAPK/ERK pathway, a specific signaling axis that is modulated by cell-matrix interactions and may therefore be influenced by culture dimensionality^10^. These differences in drug mechanisms may explain why matched 2D and 3D cultures respond similarly to classical cytostatics but can diverge in their response to targeted inhibitors. Independently, other groups previously investigated whether PDOs cultured in matched 3D Matrigel and 3D Cultrex cultures exhibit distinct transcriptional profiles. In this context, Lumibao et al. reported no significant transcriptomic differences^20^. This is notable because transcriptional subtypes in PDAC have previously been associated with differential responses to chemotherapeutic regimens such as mFOLFIRINOX and gemcitabine plus nab-paclitaxel^34^. Yet, specific signalling pathways might still be affected.

Moreover, inter‑patient variability emerged as a major determinant of drug response, with significant differences observed for 5‑FU, SN‑38 and gemcitabine. These findings underscore the need to consider intertumoral heterogeneity when interpreting in vitro therapy responses, regardless of cell culture dimensionality. This observation mirrors the clinical situation, where response rates to standard regimens such as to gemcitabine or mFOLFIRINOX vary considerably between patients^34^. Yet, there are no clinically implemented methods to predict patient specific responses. Previous studies demonsted the predictive potential of PDOs in PDAC^10,14^. Clinical follow-up data were available for two therapy-naïve patients in our study cohort. In vivo response proved to be in concordance with the relative in vitro drug response to gemcitabine. This suggests 2D and 3D in vitro models to complement individualized treatment decisions. To further investigate relative potential resistance mechanisms in these two models, MDR1 expression was assessed. MDR1 (ABCB1/P-glycoprotein) is an ATP-dependent efflux transporter that reduces intracellular drug accumulation and elevated expression in PDAC has been described as a negative prognostic factor for response to gemcitabine^35–37^. The markedly elevated MDR1 expression observed in the gemcitabine-resistant model derived from Patient 2, compared to the clinically responsive Patient 3, supports the notion that enhanced drug efflux represents a relevant resistance mechanism to gemcitabine in this case. Due to the limited number of clinically correlated cases, however, these observations must be interpreted with caution and remain purley descriptive in nature.

The limitations of the study presented include the small number of matched patient‑derived models, the absence of multi‑omics analyses and the limited number of cases for clinical correlation. Among these, the most significant limitation is the relatively small number of patient‑derived models in all culture conditions (*n*= 3). This is similar to previous organoid-based studies, with sample sizes ranging from 8 to 66^17,18,24,29^. In these studies, however, PDOs were typically established in only one rather than three culture conditions, either in Matrigel or Cultrex and not in 2D^17,18,24,29^. Genomic profiling was performed only for the PDOC cultures and not for the corresponding PDOM, PDCL, or the original FFPE tumor tissue, limiting a more comprehensive assessment of clonal selection and genomic concordance across the different culture systems. In addition, our experiments did not include multi‑omics analyses. Although we observed concordance between in vitro drug response and the clinical course in two patients receiving adjuvant chemotherapy, the small number of evaluable cases does not allow a robust assessment of clinical correlation. Nevertheless, this concordance suggests that even a limited set of patient‑derived in vitro cultures can yield meaningful insights into individual therapy responses with MRD1 expression providing one explaination to gemcitabine resistance.

In conclusion, our findings show the most commonly used matrix compositions in the field of PDAC (Matrigel vs. Cultrex) to have minimal impact on growth kinetics, while culture dimensionality influences proliferation and phenotypic marker expression. Drug responses to standard chemotherapeutics were predominantly patient‑specific and only marginally affected by culture format or matrix conditions, indicating that their efficacy appears largely independent of these cell culture variables. Notably, 3D cultures more closely recapitulated the morphological features of the original tumors, underscoring their superior biological relevance. Taken together, these results highlight that while responses to standard chemotherapies appear robust across different culture systems, 3D models offer a more realistic representation of the tumor architecture and should therefore be prioritized when modeling PDAC in vitro.

## Materials and methods

### Ethical approval and patient recruitment

Tumor samples were obtained from patients undergoing surgical resection for suspected PDAC at the Charité Universitätsmedizin Berlin and Waldfriede Hospital Berlin in 2023. Written informed consent was obtained from all participants in accordance with the Declaration of Helsinki and institutional regulations. The study was approved by the institutional ethics committee (EA1/157/21). Tumor tissue from 16 patients was used to generate three parallel culture systems per resected sample: PDCL, PDOC and PDOM. All experiments were performed in accordance with relevant guidelines and regulations.

### Tumor processing and culture establishment

Fresh tumor tissue was processed within 15 min of arrival. Samples were minced using sterile scalpels and enzymatically digested for 1.5 to 3 h in a solution containing 100 µg/mL DNase I (VWR, USA), 100 µg/mL Dispase (STEMCELL Technologies, Canada), 125 µg/mL Collagenase II (Sigma-Aldrich, Germany), 1:2000 Y-27,632 ROCK inhibitor (Abmole Bioscience, USA) and 1:200 Amphotericin B (Sigma-Aldrich, Germany). After digestion, the cell suspension was filtered through a 100 μm sterile mesh and treated with red blood cell lysis buffer (Miltenyi Biotec, Germany) for 10 min. The resulting cell pellet was divided into three equal fractions. The first fraction was seeded into T25 flasks for two-dimensional (2D) culture (PDCL). PDCL cultures were maintained and passaged upon reaching 80% confluency. Fibroblast depletion was performed between passages 1 and 4 using anti-fibroblast microbeads (Miltenyi Biotec, Germany), following the manufacturer’s instructions. The remaining two fractions were embedded in basement membrane extracts, with one seeded into Cultrex reduced growth factor (RGF) BME Type 2 domes (PDOC; R&D Systems, USA) and the other into Matrigel RGF domes (PDOM; Corning, USA), using 30 µL per dome. Culture medium^17^ was added after 15 min for Matrigel and after 30 min for Cultrex domes. Organoids were passaged when organoids exceeded 200 μm in diameter using TrypLE (Thermo Fisher Scientific) and reseeded at a 1:2 ratio in 30 µL matrix domes. Amphotericin B was included in all media for the first seven days to prevent contamination. Cultures were maintained with medium changes every 3–4 days. Mycoplasma contamination was excluded using a commercial detection kit (Applied Biological Materials, Canada).

### DNA isolation and genetic sequencing

DNA was isolated from PDOC organoids. After matrix dissolution with Cell Recovery Solution (Corning, USA) and PBS washing, cell pellets were stored at -80 °C. DNA extraction was performed using the AllPrep DNA/RNA Mini Kit (Qiagen, Germany) following the manufacturer’s protocol. In short, cell pellets were lysed in RLT Plus buffer containing β-mercaptoethanol and the lysate was loaded onto AllPrep DNA spin columns. After centrifugation, the columns were washed with AW1 and AW2 buffers and genomic DNA was eluted using prewarmed EB buffer following a brief incubation at room temperature.

All DNA samples were analyzed using hybrid capture-based next-generation sequencing technology (Agilent XT HS2) with the SureSelect Cancer Comprehensive Genomic Profiling Assay (CGP Assay, Agilent Technologies). Library preparation was fully automated on a Magnis NGS Prep System (Agilent Technologies). Sequencing was performed on a NovaSeqX Plus (Illumina). For the mutation analysis, the data from the focused DNA NGS were evaluated with the DRAGEN-Bio-IT platform (Illumina) and variants with an allele frequency of at least 3% were considered. The calculation of the tumor mutation burden was based on Chalmers et al^38^. and included all synonymous and non-synonymous, presumably somatic variants with an allele frequency of at least 3%. To exclude potential germline variants, all variants were compared with entries in the “Database of Single Nucleotide Polymorphisms (dbSNP)” and the “Genome Aggregation Database (gnomAD)” and were filtered out if necessary. In addition, known driver mutations greater than 100 entries in “COSMIC, The Catalog Of Somatic Mutations In Cancer” were not included in the calculation.

### Assessment of morphology

For morphology assessment, PDCL, PDOC and PDOM cultures were imaged daily for seven days using brightfield-confocal microscopy (ASFA^®^ SCANNER High-Content Imaging System) under standardized optical conditions. Morphological parameters were assessed descriptively. Imaging was performed using a 4 × magnification with a CCD resolution of 2200 × 2200 pixels and a pixel size of 4.5 μm. For PDCL cultures, 9 images per sample were taken at 50 μm z-intervals, the percentage confluency was assessed, along with observations on whether cells grew in clusters or were evenly distributed. It was also noted whether most cells were attached to the flask surface or floating. Cell morphology was described in terms of shape (elongated vs. round), size (small vs. large) and morphological heterogeneity with regard to both size and shape. For PDOM and PDOC cultures, 9 images per sample were taken at 150 μm intervals. The resulting z-stacks were merged into a single image for each sample. Structures were classified as either cystic or dense organoids. Their average size, size variability and confluency (defined as the percentage of the imaged area occupied by organoids) were evaluated descriptively. In addition, organoid diameters were quantified at day 3 and day 7 by measuring all visible PDOs within a standardized field of view (2124 μm × 2124 μm) in the merged images.

### Histology and Immunohistochemistry

In vitro models were fixed in 4% paraformaldehyde (PFA) at 4 °C for 1 h, washed in PBS and embedded in histogel (Thermo Fisher Scientific, Waltham, MA, USA). For organoids, the medium was removed and 500 µL PFA was added per well. Following fixation and PBS washing, organoids were embedded in histogel. All histological and immunohistochemical analyses were conducted at the Institute of Pathology, Charité Campus Mitte, using established protocols and the following antibodies: HE (Tissue Tek Prisma Plus Automated Slide Stainer, SAKURA), p53 (DO-7, Dako, 1:50), SMAD4 (EP618Y, abcam, 1:200), GATA6 (Q92908, R&D Systems, 1:100), CDX2, CK19 (RCK108, BioGenex, 1:100), CA19-9 (1116-NS-19-9, Dako, 1:500), Ki-67 (MIB-1, Dako, 1:50) and Vimentin (V9, Dako, 1:5000) (immunostaining with BenchMark XT, Ventana Medical Systems, Tucson, AZ) and MDR1 (LSBio, LS-B5570, clone JSB-1, 1:50). GATA6 was scored on a semi-quantitative scale from 0 to 4. Vimentin was evaluated qualitatively. Ki-67, CK19, CDX2 and CA19-9 were quantified as the percentage of positively stained cells. p53 and SMAD4 staining patterns were categorized as wild-type or aberrant expression patterns (loss/overexpression). MDR1 expression was assessed by calculating H-scores^39^.

For correlation analysis between proliferation and organoid size, organoid diameters were measured in Ki-67-stained scans of PDOC and PDOM cultures using QuPath (version 0.6.0). For each of the four patients, ten organoids per culture condition were analyzed, resulting in a total of 80 quantified organoids. Ki-67 expression was quantified as the percentage of positively stained nuclei per organoid using QuPath-based cell detection. Data from all patients and culture conditions were pooled, and the association between organoid diameter and Ki-67 expression was assessed using Spearman correlation analysis.

### Assessing growth kinetics

Growth kinetics were evaluated by assessing Time To First Split (TTFS) and Doubling Time (DT). TTFS was recorded in days and defined as the interval from tumor tissue acquisition (surgical resection or biopsy) to the first passage of the corresponding culture.

Growth assays were conducted between passages 3 and 15. Organoids were dissociated into single cells using TrypLE and counted via acridine orange/propidium iodide staining with an automated cell counter (LUNA™ Automated Cell Counter; BioCat, Heidelberg, Germany). Only suspensions with a single-cell ratio of at least 90% were used for seeding. For PDCL models, 7,000 cells per well were seeded into 96-well plates, while for PDOC and PDOM, 2,000 cells per well were embedded in 6 µL matrix domes in 96-well plates. A ROCK inhibitor (1:1000) was added to the culture medium for all models for all 7 days. Viability was assessed as an endpoint at each time point using the CellTiter-Glo^®^ Luminescent Assay (Promega, Germany). Cells were plated on day 0 and endpoint measurements were taken after 7 days in duplicate. Cell viability was assessed using the CellTiter-Glo^®^ assay (Promega) according to the manufacturer’s protocol. Luminescence was recorded with the VICTOR Nivo™ Multimode Microplate Reader (PerkinElmer, Germany). The mean of two technical replicates was calculated and luminescence values from days 2 to 7 were normalized to day 1. These normalized values were used to calculate doubling times (DT) and to generate growth curves.

### Pharmacotyping

Drug response assays were performed between passages 3 and 15. PDCLs, PDOCs and PDOMs models were seeded in analogy to Sect. 2.6. ROCK inhibitor (1:1000) was initially added to media and maintained for the first 96 h. Drug treatment was initiated after 96 h using a 10-fold dilution series. Maximum concentrations were as follows: SN-38 (TargetMol) and Paclitaxel (TargetMol): 10 µM; Gemcitabine (TargetMol): 50 µM; Oxaliplatin (Selleckchem): 100 µM; 5-FU (Sigma-Aldrich): 1 mM. Solvent controls matched the highest drug concentration in each dilution. Cell viability was assessed 72 h post-treatment using the CellTiter-Glo^®^ Luminescent Assay (Promega, Germany) according to the manifactures protocol. Luminescence was recorded using the VICTOR Nivo™ Multimode Microplate Reader (PerkinElmer, Germany). For each condition, three technical replicates were averaged and normalized to untreated controls. Based on the resulting percentage viability values, dose–response curves were generated and the Area Under the Curve (AUC) and IC₅₀ values were calculated by nonlinear regression. GR₅₀ values were calculated for patients 1–4 using the GRcalculator^40^, incorporating patient-specific doubling times. For non-measurable GR_50_ values, we assigned cut offs of + 3 (representing no measurable response within tested concentration; flat curve) and -4 (representing extreme sensitivity; response below tested range). To evaluate condition-dependent differences in drug response, AUC and log_10_GR_50_ values were pooled and compared between culture systems. Additionally, inter-patient variability in drug response was assessed by pooling pharmacotyping values by patient. To enable clinical comparison between patients based on relapse, relapse was defined here as the reappearance of the same tumor disease following curatively intended therapy.

### Patient follow-up

All patients enrolled in this study were monitored from the date of initial resection or biopsy until their most recent assessment. The first patient was enrolled in January 2023 and last follow-up was in May 2025. Follow‑up information comprised the date of initial diagnosis, date of resection or biopsy, overall survival, disease progression, recurrence and administration of chemotherapy.

### Systematic literature search on matrix usage in PDAC PDO studies

The following search query was applied: (“pancreatic ductal adenocarcinoma” OR PDAC OR “pancreatic cancer”) AND (organoid* OR tumoroid*). The search was restricted to publications from the last 10 years, English language, and human studies. Non-original publication types, including reviews, systematic reviews, meta-analyses, editorials, comments, guidelines, letters, case reports, preprints, and retracted publications, were excluded. Study selection was conducted in three stages: title screening, abstract screening, and full-text assessment. Studies were included if they reported patient-derived establishment of human PDAC organoids derived from primary patient tumor material (e.g., resection or biopsy specimens) and described 3D epithelial organoid cultures. Studies were excluded if they used exclusively murine models, relied solely on previously established organoid lines without new derivation, focused primarily on co-culture or tumor microenvironment reconstruction systems incorporating stromal or immune components, or described spheroid or 2D culture models. For all eligible studies, the extracellular matrix used for organoid embedding was extracted from the Methods section and categorized as Matrigel, Cultrex/basement membrane extract (BME), both, other matrix, or not stated.

### Statistics

Statistical analyses were performed using GraphPad Prism version 10.5.0 (GraphPad Software, San Diego, CA, USA). Depending on data distribution and experimental design, unpaired two-tailed t-tests, Mann–Whitney U tests, or one-way ANOVA with Tukey’s post hoc test were used for group comparisons. Normality was assessed using the Shapiro–Wilk test. For immunohistochemistry, marker expression values were pooled according to FFPE tissue or in vitro condition and compared using one-way ANOVA. Time To First Split (TTFS) across all cultures from the 12 included patients was compared between in vitro conditions using one-way ANOVA with Tukey’s post hoc test. Correlations between doubling time and Ki-67 expression in vitro, were analyzed using linear regression. Results were considered statistically significant at *p* < 0.05. Data are presented as mean ± standard deviation (SD), unless otherwise specified. All experiments were conducted in at least two technical replicates.

## Supplementary Information

Below is the link to the electronic supplementary material.

Supplementary Material 1

## Data Availability

The sequencing data generated in this study have been deposited in the European Nucleotide Archive (ENA) with the accession number PRJEB97488.
