# Supplementary material for: Impact of culture dimensionality and matrix composition on morphology, phenotype and drug response in pancreatic cancer models
Source: Sci Rep. 2026 Apr 14;16:12346. doi: 10.1038/s41598-026-47856-1 (PMC13079817; doi:10.1038/s41598-026-47856-1)
Supplement: Supplementary file 1 — Supplementary Material 1 [file 41598_2026_47856_MOESM1_ESM.docx]

***Supplementary Figure S1. Correlation of organoid size and Ki-67 expression in PDAC PDOs.***

*Scatter plot showing the association between PDO diameter and Ki-67. PDO diameters were measured from Ki-67-stained histological scans, Ki-67 expression was quantified as the percentage of Ki-67-positive nuclei per organoid. In total, 80 organoids derived from four patients and cultured under both matrix conditions were included in the analysis. Organoid diameters ranged up to approximately 400 µm. Spearman correlation analysis revealed no statistically significant association between organoid diameter and Ki-67 expression (r = −0.19, p = 0.087).*

*
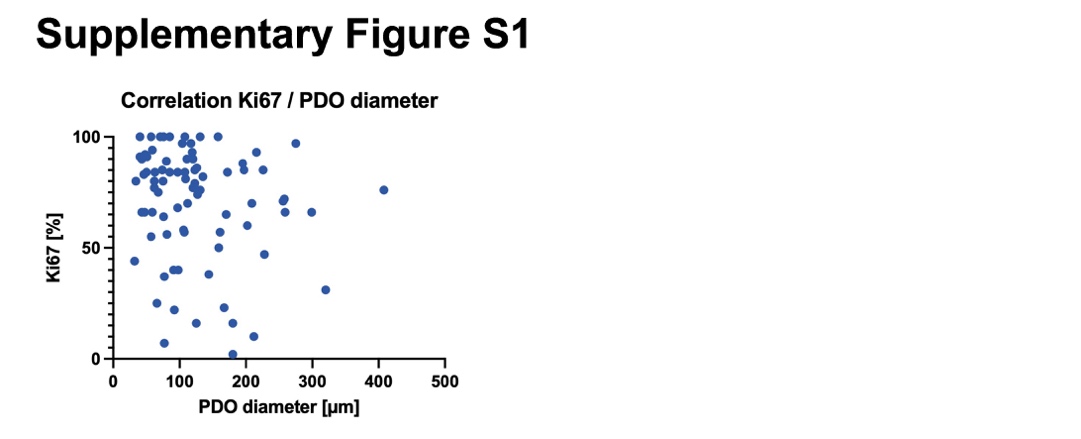
*

***Supplementary Figure S2. Patient-specific drug response profiles in PDAC cultures across three in vitro models.***

*Dose-response curves for five chemotherapeutic agents - 5-fluorouracil (5-FU), SN-38 (active metabolite of irinotecan), oxaliplatin, gemcitabine and paclitaxel - across PDCL (blue, triangles), PDOC (brown, circles) and PDOM (beige, squares) models derived from four PDAC patients (A-D). Each curve represents a single culture model for the respective patient. Cell viability was assessed after 72 hours of drug exposure using luminescence-based assays. Data represent mean values from technical replicates.*

*
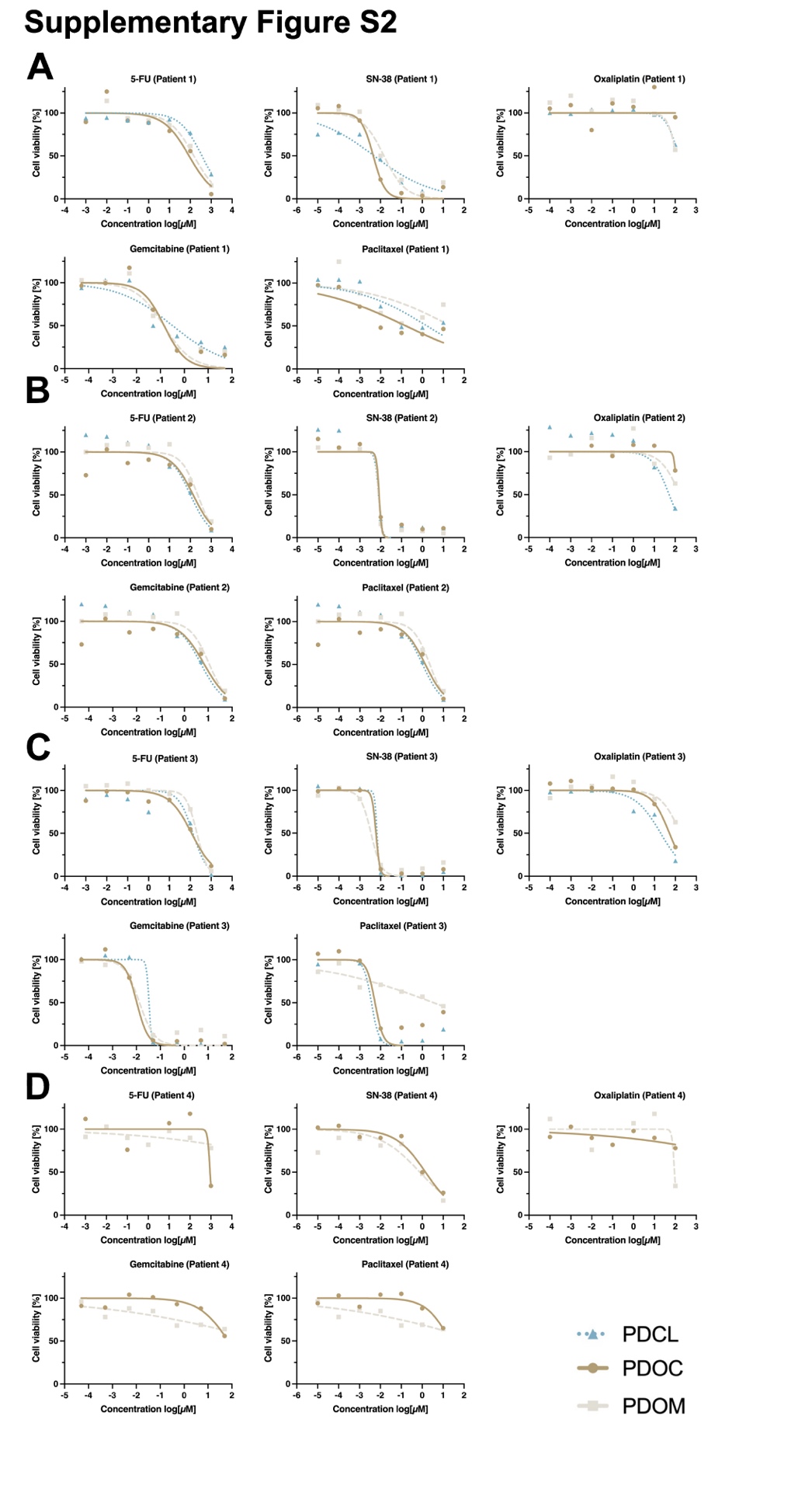
*

***Supplementary Table S1. Histological assessment of morphology and desmoplasia***

*Histopathological characteristics of the original FFPE tumor specimens from four PDAC patients, including tumor morphology, grading, and the extent of desmoplasia according to the classification described by Schmid et al.. In addition, morphological features of the corresponding patient-derived in vitro models are summarized, including PDOC, PDOM and PDCL. The table highlights similarities and differences in architectural growth patterns between the primary tumor tissue and the respective in vitro culture systems.*

|  | Patient 1 | Patient 2 | Patient 3 | Patient 4 |
| --- | --- | --- | --- | --- |
| Morphology FFPE | gland forming, focal confluent glands, G2 (grade) | gland forming, G2 (grade) | solide growth patter, with tumor nests, G3 | gland forming, G2 (grade) |
| Desmoplasia FFPE | Grade I = less than 50% of surface covered by desmoplastic tissue | Grade I = less than 50% of surface covered by desmoplastic tissue | Grade II = 50–75% of the tumour contains desmoplastic tissue | Grade I = less than 50% of surface covered by desmoplastic tissue |
| Morphology PDCL | disorganized strands of cells, very few glands | small clusters of cells, no gland formation | small nests and single cells | not established |
| Morphology PDOC | large and small single layered glands | small, double layered glands | nests with cribriform growth | small monolayerd glands and few multilayerd nodes |
| Morphology PDOM | small multilayered glands and few large single layered glands | medium and large monolayerd glands | small cell nests | medium cribriform glands and large monolayered glands |

|  | Sample Type | p53 pattern | SMAD4 pattern | Ki-67 (%) | GATA6 Score | CK19 (%) | CDX2 (%) | CA19-9 (%) | Vimentin |
| --- | --- | --- | --- | --- | --- | --- | --- | --- | --- |
| 1 | FFPE | mut. (loss) | mut. (loss) | 35% | 3 | 100% | 50 | 100% | focal |
| 1 | PDOC | WT | WT | 70% | 3 | 100% | 75 | 95% | focal weak |
| 1 | PDOM | WT | WT | 80% | 3 | 100% | 75 | 50% | focal very few |
| 1 | PDCL | WT | WT | 50% | 3 | 100% | 75 | 80% | focal very few |
| 2 | FFPE | WT | mut. (loss) | 20% | 2 | 100% | 50 | 100% | negative |
| 2 | PDOC | WT | WT / mut. | 95% | 4 | 100% | 90 | 100% | negative |
| 2 | PDOM | WT | WT / mut. | 85% | 4 | 100% | 90 | 100% | focal very few |
| 2 | PDCL | WT | WT/ mut. | 40% | 4 | 100% | 95 | 100% | focal very few |
| 3 | FFPE | mut. (OE) | mut. (loss) | 10% | 3 | 100% | 70 | 10% | negative |
| 3 | PDOC | mut. (OE) | WT / mut. | 70% | 4 | 100% | 95 | 0% | negative |
| 3 | PDOM | mut. (OE) | WT / mut. | 75% | 4 | 100% | 95 | 0% | focal very few |
| 3 | PDCL | mut. (OE) | WT / mut. | 70% | 4 | 100% | 95 | 0% | focal very few |
| 4 | FFPE | mut. (OE) | mut. (loss) | 15% | 3 | 100% | 5 | 70% | negative |
| 4 | PDOC | mut. (OE) | WT | 35% | 3 | 100% | 50 | 90% | focal very few |
| 4 | PDOM | mut. (OE) | WT | 30% | 4 | 100% | 40 | 90% | negative |

***Supplementary Table S2. Immunohistochemical marker expression in FFPE tumor tissue and corresponding patient-derived culture models.***

*Expression of diagnostic and phenotypic markers was assessed by immunohistochemistry in formalin-fixed paraffin-embedded (FFPE) primary tumor samples and in matched 2D monolayer cultures (PDCL) and 3D organoid cultures embedded in Cultrex (PDOC) or Matrigel (PDOM). Ki-67, CK19, CDX2 and CA19-9 were quantified as the percentage of positively stained cells. GATA6 expression was evaluated on a 0-4 scoring scale. Vimentin expression was qualitatively reported as negative or focal, based on intensity and distribution. p53 and SMAD4 staining patterns were categorized as wild-type (WT) or aberrant expression patterns (loss or overexpression, OE).*
